# Predictive value of *DOT1L* mutations for clinical outcomes in non‐small‐cell lung cancer patients receiving immune checkpoint inhibitor therapy

**DOI:** 10.1002/ctm2.1430

**Published:** 2023-10-02

**Authors:** Tianxing Guo, Zhaofeng Wang, Song Wang, Yaru Zhang, Jiaohui Pang, Yajun Ying, Qiuxiang Ou, Dong Shen, Gang Li

**Affiliations:** ^1^ Department of Thoracic Surgery Shengli Clinical Medical College of Fujian Medical University Fujian Provincial Hospital Fuzhou China; ^2^ Department of Respiratory Medicine Affiliated Jinling Hospital, Medical School of Nanjing University Nanjing China; ^3^ Geneseeq Research Institute Nanjing Geneseeq Technology Inc. Nanjing China; ^4^ Department of Pathology Taizhou Cancer Hospital, Taizhou Zhejiang China; ^5^ Department of Oncology The Affiliated Jiangyin Hospital of Nantong University, Jiangyin Jiangsu China; ^6^ Department of Thoracic Surgery Sichuan Provincial People's Hospital, University of Electronic Science and Technology of China Chengdu China; ^7^ Department of Thoracic Surgery Sichuan Translational Medicine Research Hospital, Chinese Academy of Sciences Chengdu China

Dear Editor,

In this study, we present findings that highlight the role of baseline Disruptor of telomeric silencing 1‐like (*DOT1L*) mutations as a promising biomarker to identify stage IIIb/IV non‐small‐cell lung cancer (NSCLC) patients who might demonstrate potential clinical benefit under immune checkpoint inhibitor (ICI) therapies.

NSCLC patients often present advanced/metastatic disease upon diagnosis. While ICI therapy has demonstrated effectiveness, major limitations include heterogeneous responses among patients, acquired resistance, and immune‐related adverse effects.^1^ Therefore, it is crucial to identify reliable predictive biomarkers for therapy efficacy beyond programmed cell death protein 1 (PD‐1) expression and tumor mutation burden (TMB) for patient stratification as well as precision medicine. Accumulating evidence has suggested that epigenetic aberrations are key drivers of cancer initiation, progression, and therapeutic resistance.^1^ DOT1L is the sole histone lysine methyltransferase that operates in epigenetic modulation of gene expression by methylating the lysine 79 residue on histone H3 (H3K79).^2^ While being first reported in mixed lineage leukemia (MLL)‐rearranged leukemia, DOT1L overexpression has been linked to unfavorable clinical outcomes in various nonhematologic neoplasms, including estrogen receptor α‐positive breast cancer, ovarian cancer, prostate cancer, gastric cancer, colorectal cancer, and clear‐cell renal cell carcinoma.^2^ Notably, Noblejas‐López et al. have shown that DOT1L is an indicator of eligibility for immunotherapy in breast cancer patients.^3^ In lung adenocarcinoma (ADC), *DOT1L* mutations were found in ∼3% of cases.^4^ The downregulation of *DOT1L* in lung cancer cells reduced H3K79 methylation levels, which was associated with disturbed cell proliferation, as cells were arrested in the G1 phase and displayed chromosomal segregation abnormalities.^5^ However, the reduction of H3K79me3 during the transforming growth factor beta 1 (TGF‐β1)‐induced epithelial‐to‐mesenchymal transition (EMT) in lung cancer was found independent of DOT1L expression; and both DOT1L inhibitors (EPZ5676 and SGC0946) partially reversed the increased expression of EMT‐related genes.^6^ In a preclinical study, Scheer et al. reported that DOT1L‐dependent H3K79me2 has been associated with the functional differentiation of CD4+ T helper cells and anti‐tumor immune response under both lineage‐specific and ‐promiscuous conditions.^7^ Collectively, these findings imply a complex regulatory role of DOT1L‐mediated H3K79 methylation. More studies are required to establish the link between the functional significance of DOT1L and its clinical implications in NSCLC patients, particularly those who underwent ICI therapy.

Here, we conducted comprehensive genomic analyses using primary tumor samples from 393 patients diagnosed with stage III/IV NSCLC (the study cohort), along with data from three external cohorts as follows: (1) the NSCLC‐ICI cohort, including 349 NSCLC patients treated with ICIs; (2) the MSK‐ICI cohort, including 1661 patients with various solid tumors subjected to ICI therapy; (3) The Cancer Genome Atlas (TCGA)‐NSCLC cohort, including 994 NSCLC patients with clinical and mRNA expression data (Figure 1A, Supplementary Methods). In our study cohort, most patients were male (61.3%) and over 60 years of age (59.8%), with ADC being the predominant histological type (87.3%, 343/393) (Table 1). Consistent with a previous report indicating that *DOT1L* mutations occurred in 3% of ADC patients,^4^ our study found *DOT1L* mutations in 3.6% of all cases. Among these mutations, the predominant type was missense mutations (62.5%, 10/16), followed by frameshift variants (31.3%, 5/16). Only one nonsense mutation was found, and G137R was identified as the sole cancer hotspot in tumor samples from the study cohort (Figure S1A; Table S1). Additionally, *DOT1L* mutations were more frequent in males (*p* = .001) and patients with non‐ADC compared to ADC (8% vs. 2.9%, *p* = .088) (Figure S1B,C).

**FIGURE 1:**
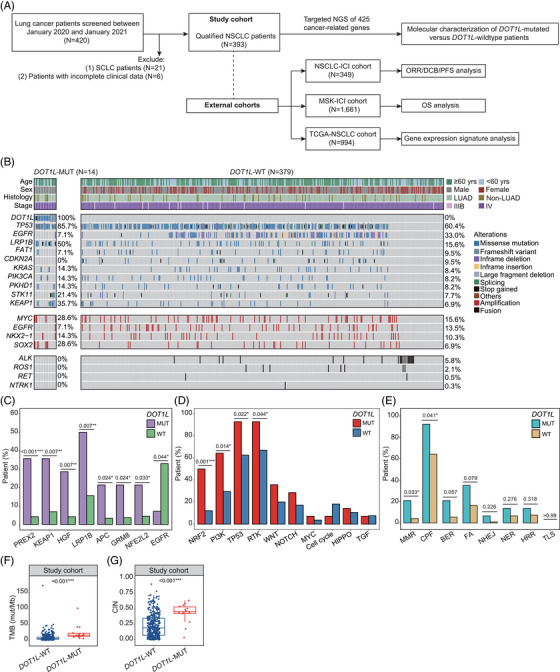
Mutational landscape of non‐small‐cell lung cancer (NSCLC) patients harboring *DOT1L* mutations. (A) An overview of the study design. The study cohort comprises 393 NSCLC patients screened at all participating hospitals between January 2020 and January 2021. Three independent external datasets (see Supplementary Material for details) were assessed to investigate the clinical implications of *DOT1L* mutations, including the NSCLC‐immune checkpoint inhibitor (ICI) cohort (*N* = 349) for assessing the relationship between *DOT1L* mutations and short‐term clinical outcomes, the MSK‐ICI cohort (*N* = 1661) for overall survival, and The Cancer Genome Atlas (TCGA)‐NSCLC cohort for exploring mRNA expression signatures associated with *DOT1L*‐mutated patients. (B) Co‐mutation plot depicting NSCLC patients with or without *DOT1L* mutations. Each column represents one patient. (C) The bar plot of patient percentage harboring specific concurrent genetic alterations between *DOT1L*‐mutated and *DOT1L*‐wildtype patients. (D) The bar plot of patient percentage with an altered oncogenic pathway between the two patient subgroups. (E) The bar plot of patient percentage with DNA damage response (DDR)‐related pathway gene aberrations. (F and G) Box plots illustrating the TMB (F) and chromosomal instability (CIN) score (G) between subgroup patients in the study cohort. MMR, mismatch repair; CPF, checkpoint factor; BER, base excision repair; FA, Fanconi anemia; NHEJ, non‐homologous end join; NER, nucleotide excision repair; HRR, homologous recombination repair; TLS, translesion synthesis.

**TABLE 1:** Clinical characteristics of patients in the discovery cohort (*N* = 393).

Characteristic	All (*N* = 393)	*DOT1L*‐WT (*N* = 379)	*DOT1L*‐MUT (*N* = 14)	*p*‐Value
Sex				.001
Female	152 (38.7%)	152 (40.1%)	0 (.00%)	
Male	241 (61.3%)	227 (59.9%)	14 (100%)	
Age				.79
<60	158 (40.2%)	153 (40.4%)	5 (35.7%)	
≥60	235 (59.8%)	226 (59.6%)	9 (64.3%)	
Median (range)	63 (26‐90)	63 (26‐90)	71 (55‐84)	
Clinical stage				>.99
IIIb	14 (3.56%)	14 (3.69%)	0 (.00%)	
IV	379 (96.4%)	365 (96.3%)	14 (100%)	
Histology				.18
ADC	343 (87.3%)	333 (87.9%)	10 (71.4%)	
SCC	45 (11.5%)	41 (10.8%)	4 (28.6%)	
ASC	4 (1.02%)	4 (1.06%)	0 (.00%)	
PSC	1 (.25%)	1 (.26%)	0 (.00%)	

*Note*: *p*‐Values are based on Fisher's exact test.

Abbreviations: ADC, adenocarcinoma; ASC, adenosquamous cell carcinoma; PSC, pulmonary sarcomatoid carcinoma; SCC, squamous cell carcinoma.

The most frequently mutated gene in our study cohort was *TP53* (Figure 1B). While *DOT1L* mutations were likely to co‐occur with aberrations in *PREX2*, *KEAP1*, *HGF*, *LRP1B*, *APC*, *GRM8*, and *NFE2L2* genes, only one *DOT1L*‐mutated patient had a concomitant deletion mutation in exon 19 of *EGFR* (Figure 1B, C). Meanwhile, *DOT1L* mutations were significantly enriched in patients with altered NRF2, PI3K, TP53, RTK, and DDR‐related pathways, specifically the mismatch repair (MMR) pathway and the checkpoint factor (CPF) pathway (Figure 1D,E). Moreover, we observed distinct patterns while examining the mutual exclusivity and co‐occurrence of the top 30 mutated genes in subgroup patients (Figure S1D). Interestingly, patients with baseline *DOT1L* mutations showed significantly higher TMB and chromosomal instability (CIN) scores compared to those without the mutation (both *p* < .001) (Figure 1F,G; Figure S1E). However, neither the microsatellite instability (MSI) score nor the MSI/MSS (microsatellite stable) distribution in patients exhibited significant differences between the two subgroups, presumably due to the limited sample size (Figure S1F,G). Previous studies have highlighted the role of DOT1L‐mediated H3K79 methylation in regulating cell cycle progression across various organisms, including yeast, trypanosome, mice, and human lung cancer cells.^5^ Notably, *DOT1L* deficiency in lung cancer cell lines induces cellular senescence and chromosomal instability, halting cell proliferation through G1 phase cell cycle arrest.^5^ This finding may provide a plausible explanation for the escalated CIN number seen in *DOT1L*‐mutated tumors. However, the involvement of baseline *DOT1L* mutations in promoting higher TMB and chromosomal instability still necessitates further investigation.^2^

Given the survival benefits conferred by ICIs in advanced NSCLC, our subsequent focus was to explore the functional significance of *DOT1L* mutations within the NSCLC‐ICI cohort (Figure 1A). Consistent with the findings in our study cohort, *DOT1L* mutations were present in approximately 3.72% of the NSCLC‐ICI cohort (Table S2; Figure S2A). Remarkably, patients harboring *DOT1L* mutations demonstrated a notably higher rate of durable clinical benefit (DCB; *p* = .018) and objective response rate (ORR; *p* < .001) compared to individuals lacking the mutation (Figure 2A,B). Consistently, the progression‐free survival (PFS) of patients harboring baseline *DOT1L* mutations was significantly longer than those without the alteration (median: 21.1 vs. 3.9 months, *p* = .006) (Figure 2C). Additionally, *DOT1L*‐mutated patients exhibited longer PFS than *DOT1L*‐wildtype patients in both treatment groups, and the difference in survival benefit reached statistical significance in subgroup patients receiving combination therapy (*p* = .016) (Figure 2D). Besides, we found that *DOT1L*‐mutated patients who underwent combination therapy had a significantly prolonged PFS than those treated with monotherapy (*p* = .022), suggesting that ICI‐combined chemotherapy might be the optimal treatment strategy for advanced NSCLC patients harboring *DOT1L* mutations. We also noticed a longer PFS in *DOT1L*‐mutated patients than *DOT1L*‐wildtype patients, regardless of their TMB status (Figure 2E; Figure S3A). While due to the small sample size, the observed difference was not statistically significant. The positive correlation between *DOT1L* mutations and better patient survival was not affected by baseline *EGFR* or *ALK* driver alterations (Figure 2F,G). Despite several risk factors, including *DOT1L* mutational status, smoking history, treatment, PD‐L1, and TMB status, being independently associated with prognosis at multivariate analysis (Table S3), the risk score that combines all these contributing factors was found to be effective in selecting patients who could potentially benefit from ICI treatment (Figure 2H,I; Figure S3B,C).

**FIGURE 2:**
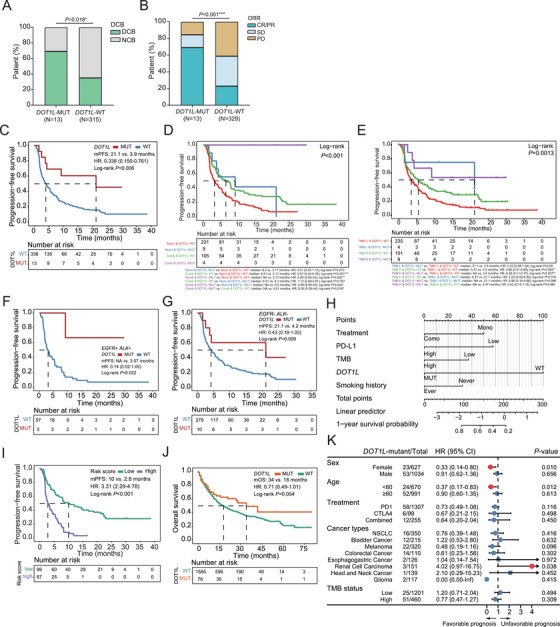
Baseline *DOT1L* mutations predict a better clinical response and survival followed by immune checkpoint inhibitor (ICI) treatment. (A) Bar plot illustrating the distribution of non‐small‐cell lung cancer (NSCLC) patients receiving ICI treatments who had durable clinical benefit (DCB) and no clinical benefit (NCB) within two subgroup patients classified based on *DOT1L* mutational status. (B) Bar plot illustrating the distribution of patients with different overall response rates (ORR) between two subgroup patients. (C) Kaplan–Meier curves of the progression‐free survival (PFS) in *DOT1L*‐wildtype versus *DOT1L*‐mutant NSCLC patients receiving ICI treatments (*N* = 349). (D) Kaplan–Meier curves showing subgroup patients’ PFS based on their ICI treatment and *DOT1L* mutational status. (E) Kaplan–Meier curves demonstrating subgroup patients’ PFS based on their TMB and DOT1L status. (F and G) Kaplan–Meier curves demonstrating the PFS of NSCLC patients with *DOT1L* mutations compared to those without, with subgroup analysis based on the presence (F) or absence (G) of driver alterations in *EGFR* and *ALK* genes. (H) The monogram demonstrates the predicted 1‐year survival probability of patients generated by incorporating significant factors identified in the multivariate analysis. (I) Kaplan–Meier curves demonstrating the PFS of high‐risk versus low‐risk NSCLC patients on ICI treatment. (J) Kaplan–Meier curves of overall survival in strata of baseline *DOT1L* mutation status among patients diagnosed with solid tumors (*N* = 1661). (K) Forest plot demonstrating the relationship between *DOT1L* mutations and overall survival of subgroup patients in cancer patients receiving ICI treatments. CR, complete response; PR, partial response; SD, stable disease; PD, progressive disease.

Next, we employed the MSK‐ICI cohort (*N* = 1661) to assess whether baseline *DOT1L* mutations could correlate with patients’ overall survival (OS) in solid tumors (Figure 1A). Patients who were identified as *DOT1L* mutation‐positive exhibited a trend towards longer OS compared to those without the mutation (*p* = .054) (Figure 2J). Among NSCLC patients within the MSK‐ICI dataset, 16 (4.6%) were identified as *DOT1L*‐mutated patients (Figure S2B). Although the univariate analysis did not show a statistically significant OS benefit for *DOT1L*‐mutant NSCLC patients, a consistent trend was observed, suggesting that *DOT1L* mutations are likely indicative of a more favorable prognosis in NSCLC (Figure 2K). In patients with certain types of solid tumors, such as renal cell carcinoma, head and neck, and bladder cancers, mutations in *DOT1L* were likely associated with worse overall survival. These findings suggest that the clinical implications of *DOT1L* mutations for OS benefits may vary depending on the specific type of cancer. Additional studies using larger study cohorts are warranted to draw a definitive conclusion.

Furthermore, we explored the functional role of DOT1L in tumor development and progression using transcriptomic data from the TCGA‐NSCLC dataset (Figure 1A). Notably, 3.2% of patients within the dataset were found to harbor *DOT1L* aberrations (Figure S2C). Furthermore, a significant portion of patients within the TCGA‐NSCLC cohort exhibited likely inactivating *DOT1L* aberrations, with five patients identified to have deep deletions, three with nonsense mutations, and one with splicing variant. *DOT1L*‐mutated patients were found to have a higher proportion of activated neutrophils than those without the mutation (*p* = .015) (Figure 3A). Additionally, the expression levels of immune‐related genes, including *VISTA*, *CD70*, *PDCD1*, *TNFRSF14*, *ARG1*, and *CD39*, were significantly lower in *DOT1L*‐mutated tumors (Figure 3B). Previous studies have suggested that epigenetic events‐driven aberrant expression of immune checkpoints, including but not limited to *PDCD1* and *VISTA*, can shift the immune environment to be more suppressive, thereby enabling cancerous cells to evade the host immune recognition and immunogenicity.^1^, ^8^ Meanwhile, although high expression of TNFRSF14 and CD39 are known to confer unfavorable outcomes in various cancers,^9^, ^10^ their prognostic significance remains largely unexplored in NSCLC and requires further investigation. Furthermore, pathway enrichment analysis of differentially expressed genes showed that *DOT1L*‐mutated patients had significantly lower gene expression in leukocyte migration and neutrophil chemotaxis, which were likely essential for chemokine activity via modulating ligand‐receptor binding in the tumor microenvironment (Figure 3C,D). Notably, we also found an elevation in the expression of E2F and MYC proto‐oncogene (MYC) targets, G2M checkpoint, and PI3K/AKT/mTOR signaling in *DOT1L* mutant samples (Table S4). The concurrent up‐regulation of oncogenic pathways in *DOT1L* mutant samples is indeed a complex and intriguing phenomenon, which might involve various contributing factors like tumor heterogeneity, pathway interactions, alternative activation due to feedback effect, DOT1L‐mediated epigenetic regulation, and restricted sample size.

**FIGURE 3:**
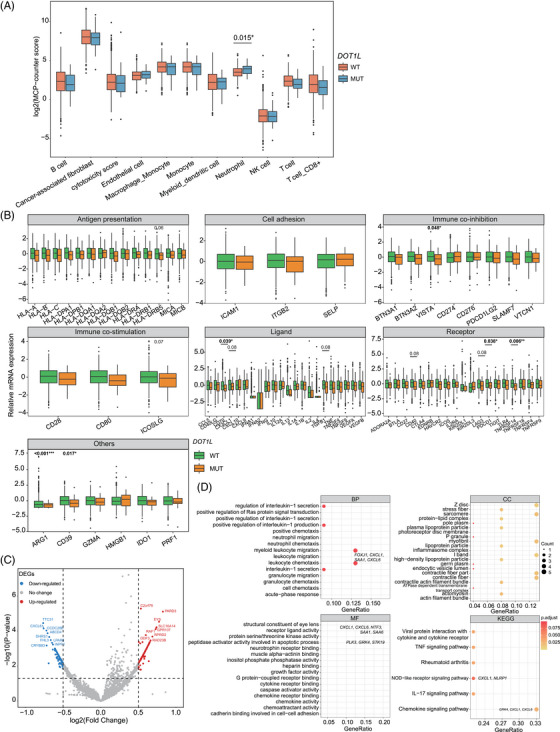
*DOT1L* mutations mitigate immune‐related gene expression in non‐small‐cell lung cancer (NSCLC). Box plot illustrating the immune cell infiltration profile of NSCLC patients in strata of *DOT1L* mutation status examined by the “MCP‐counter” approach. (B) Relative mRNA expression of immune response‐relevant genes in *DOT1L*‐mutated and *DOT1L*‐wildtype patients. (C) The volcano plot illustrates differentially expressed genes (DEGs) between *DOT1L*‐mutated and *DOT1L*‐wildtype patients. (D) Pathway enrichment analysis of significantly down‐regulated DEGs identified in *DOT1L*‐mutated patients. MCP, microenvironment cell population.

Despite these promising results, we recognize certain limitations in our study. The major drawback was the small sample size, particularly those with *DOT1L* mutations. While the current study represents pilot study results investigating the predictive role of *DOT1L* mutations for ICI treatment response, we fully recognize the importance of conducting further studies with larger cohorts to enhance the validity of our findings and provide more comprehensive insights. We are committed to pursuing such studies in the future to provide more evidence for the observations drawn from our research. Secondly, the mutation sites identified in this study for *DOT1L* mutations, along with those previously reported by others, exhibited a wide distribution, with hotspots being rarely observed. Therefore, it is imperative to acknowledge the limitation arising from the absence of detailed functional assays that would inform the biological impact of *DOT1L* mutations and provide mechanistic insights through which these mutations potentially confer improved outcomes in response to ICI treatment. Future studies that combine functional analyses would further advance our understanding of the role of *DOT1L* mutations in advanced NSCLC and guide the development of personalized therapeutic strategies. Lastly, while bulk RNA analysis offers valuable insights into the expression profile of patients with and without baseline *DOT1L* mutations, future studies that employ more comprehensive analytical approaches, such as Cleavage Under Targets and Tagmentation (CUT&Tag) and single‐cell sequencing, may provide a more detailed understanding of the association among DOT1L‐mediated epigenetic regulation, tumor heterogeneity, intricate pathway crosstalk, and ICI response in patients.

## CONCLUSION

In conclusion, our research indicated that baseline *DOT1L* mutations may serve as a predictive biomarker for ICI response in NSCLC patients, which may facilitate better patient stratification and inform optimal treatment decision‐making in clinical practice.

## CONFLICT OF INTEREST STATEMENT

SW, YRZ, JHP, and QXO are employees of Nanjing Geneseeq Technology Inc. The remaining authors declare no competing interests.

## Supporting information

Supporting Information

## Data Availability

The datasets generated and/or analyzed during this current study are available from the corresponding author upon reasonable request.
