# Supplementary material for: Predictive value of DOT1L mutations for clinical outcomes in non‐small‐cell lung cancer patients receiving immune checkpoint inhibitor therapy
Source: Clin Transl Med. 2023 Oct 2;13(10):e1430. doi: 10.1002/ctm2.1430 (PMC10545889; doi:10.1002/ctm2.1430)
Supplement: Supplementary file 1 — Supporting Information [file CTM2-13-e1430-s001.docx]

**Supplementary Information**

## Materials and Methods

### Patients and sample collection

Tumor samples were collected from 420 lung cancer patients admitted to all participating hospitals between January 2020 and January 2021. Formalin-fixed paraffin-embedded (FFPE) tumors were confirmed by pathologists from the centralized clinical testing center. This study was conducted in accordance with the declaration of Helsinki and was approved by the Ethical Committee of Sichuan Provincial People’s Hospital (No. 202274). Written consent form was obtained from each patient before sample collection.

Three independent external datasets were included in this study. Specifically, the NSCLC-ICI cohort consisted of 349 NSCLC patients treated with ICIs [1-3], and their clinical and mutational data was obtained from cBioPortal (https://www.cbioportal.org/). The evaluation of the clinical efficacy of patients was assessed by investigator-assessed Response Evaluation Criteria in Solid Tumors (RECIST) version 1.1. Besides, efficacy was categorized as durable clinical benefit (DCB), defined as complete response (CR)/partial response (PR) or stable disease (SD) that lasted >6 months, and no clinical benefit (NCB), defined as progressive disease (PD) or SD that lasted ≤6 months [1]. Progression-free survival (PFS) was defined as the duration from the initiation of ICI treatment until the time of disease progression. Patients who had not progressed were censored at the date of their last scan. The MSK-ICI cohort included 1,661 patients with different types of solid tumors who underwent ICI treatments to evaluate the impact of *DOT1L* mutations on the overall survival (OS) of patients [4]. OS was defined as the time from treatment initiation until death, regardless of disease recurrence. Patients who did not die were censored at the date of the last follow-up. The Cancer Genome Atlas-Lung Adenocarcinoma (TCGA-LUAD) and TCGA-Lung Squamous Cell Carcinoma (LUSC) cohorts (hereafter referred to as TCGA-NSCLC) were collected from the TCGA database [5] to analyze the differences in the tumor microenvironment between *DOT1L*-mutated and wildtype NSCLC patients.

### Targeted next-generation sequencing

DNA extraction, library construction, and targeted NGS were performed as previously described in a Clinical Laboratory Improvement Amendments (CLIA)-certified and College of American Pathologists (CAP)-accredited clinical testing laboratory (Nanjing Geneseeq Technology Inc., Nanjing, China) [6, 7]. Briefly, FFPE samples were de-paraffinized with xylene, followed by genomic DNA extraction using QIAamp DNA FFPE Tissue Kit (Qiagen Cat. No. 56404) according to the manufacturer’s instructions. Genomic DNA of white blood cells in sediments was extracted using the DNeasy Blood and Tissue Kit (Qiagen Cat. No. 69504) as normal control. Genomic DNA was qualified using Nanodrop2000 (Thermo Fisher Scientific, Waltham, MA), and cfDNA fragment distribution was analyzed on a Bioanalyzer 2100 using the High Sensitivity DNA Kit (Agilent Technologies, Santa Clara, CA, 5067-4626). DNA quantification was performed using the dsDNA HS assay kit on a Qubit 3.0 fluorometer (Life Technology, US). NGS libraries were prepared using the KAPA Hyper Prep kit (KAPA Biosystems) with an optimized manufacturer’s protocol for different sample types. Hybridization-based target enrichment was carried out with GeneseeqPrime^TM^ targeted NGS panel and xGen Lock-down Hybridization and Wash Reagents Kit (Integrated DNA Technologies) [8]. The target-enriched library was then sequenced on HiSeq4000 or HiSeq4000 NGS platforms (Illumina) according to the manufacturer’s instructions.

### Mutation calling

Sequencing data were first demultiplexed and subjected to FASTQ file quality control using Trimmomatic [9]. Qualified data (QC above 15 and without extra N bases) was then mapped to the human genome Hg19 using Burrows-Wheeler Aligner (BWA-mem, v0.7.12; http://github.com/lh3/bwa/tree/master/bwakit). Local realignment around the indels and recalibration of the base quality score was performed using the Genome Analysis Toolkit (GATK 3.4.0; http://software.broadinstitute.org/gatk/). Duplicates generated during sample preparation were removed using Picard. VarScan2 was applied to detect single-nucleotide variations (SNVs) and insertion/deletion mutations. SNVs were filtered out if the variant allele frequency was less than 1%. Common SNVs were excluded if they were present in >1% population in the 1000 Genomes Project or the Exome Aggregation Consortium 65,000 exomes database. The resulting mutation list was further filtered by an in-house list of recurrent artifacts based on a normal pool of whole blood samples. Parallel sequencing of matched white blood cells (control) from each patient was performed to remove sequencing artifacts, germline variants, and clonal hematopoiesis. Tumor mutation burden (TMB) was calculated as the total number of nonsynonymous mutations divided by the length of the genomic target region. TMB-high (TMB-H) tumors were defined as tumors with TMB≥10 mut/Mb, whereas TMB-low (TMB-L) tumors were defined as tumors with TMB<10 mut/Mb. In agreement with previous publications, the chromosomal instability (CIN) score was defined as the proportion of DNA segments with a log2 ratio >±0.2 in all the covered regions of the genome [10]. Variant calling, TMB, and CIN definitions were validated with CLIA/CAP accreditation.

### Immune characteristics analysis

The infiltration estimation scores of TCGA-NSCLC tumors were downloaded from the TIMER2.0 website (http://timer.cistrome.org/) [11]. Microenvironment Cell Populations-counter (MCP-counter) was used to analyze the proportion of immune and non-immune cell infiltrations using default parameters [12]. The mRNA expression level of immune-related genes [13], including antigen presentation, cell adhesion, co-inhibitor, co-stimulator, ligand, receptor, and others, was compared among subgroup patients with or without *DOT1L* mutations.

### Pathway enrichment analysis

Genes related to DNA damage repair were downloaded from the MsigDB [14]. Gene set enrichment analysis (GSEA) [15] was used to analyze and compare the variations in pathway activities derived from Gene Ontology (GO) and Kyoto Encyclopedia of Genes and Genomes (KEGG) between *DOT1L*-mutated and *DOT1L­*-wildtype groups.

### Statistical analysis

All statistical analyses were performed in R (version 4.1.3). Fisher’s exact tests were used to compare the frequencies of categorical measures among different groups, and Wilcoxon rank sum tests were used to compare the distribution of continuous data. Kaplan-Meier curves were used to analyze the PFS or OS of different patient groups, and the statistical difference was assessed using the log-rank test. A two-sided *P* value of less than 0.05 was considered significant for all tests unless indicated otherwise (**P*<0.05, ***P*<0.01, ****P*<0.001).

## References

1. N.A. Rizvi, M.D. Hellmann, A. Snyder, P. Kvistborg, V. Makarov, J.J. Havel, et al., *Cancer immunology. Mutational landscape determines sensitivity to PD-1 blockade in non-small cell lung cancer.* Science, 2015. **348**(6230): p. 124-8.

2. M.D. Hellmann, T. Nathanson, H. Rizvi, B.C. Creelan, F. Sanchez-Vega, A. Ahuja, et al., *Genomic Features of Response to Combination Immunotherapy in Patients with Advanced Non-Small-Cell Lung Cancer.* Cancer Cell, 2018. **33**(5): p. 843-852 e4.

3. H. Rizvi, F. Sanchez-Vega, K. La, W. Chatila, P. Jonsson, D. Halpenny, et al., *Molecular Determinants of Response to Anti-Programmed Cell Death (PD)-1 and Anti-Programmed Death-Ligand 1 (PD-L1) Blockade in Patients With Non-Small-Cell Lung Cancer Profiled With Targeted Next-Generation Sequencing.* J Clin Oncol, 2018. **36**(7): p. 633-641.

4. R.M. Samstein, C.H. Lee, A.N. Shoushtari, M.D. Hellmann, R. Shen, Y.Y. Janjigian, et al., *Tumor mutational load predicts survival after immunotherapy across multiple cancer types.* Nat Genet, 2019. **51**(2): p. 202-206.

5. K. Tomczak, P. Czerwinska and M. Wiznerowicz, *The Cancer Genome Atlas (TCGA): an immeasurable source of knowledge.* Contemp Oncol (Pozn), 2015. **19**(1A): p. A68-77.

6. Z. Yang, N. Yang, Q. Ou, Y. Xiang, T. Jiang, X. Wu, et al., *Investigating Novel Resistance Mechanisms to Third-Generation EGFR Tyrosine Kinase Inhibitor Osimertinib in Non-Small Cell Lung Cancer Patients.* Clin Cancer Res, 2018. **24**(13): p. 3097-3107.

7. Y. Shu, X. Wu, X. Tong, X. Wang, Z. Chang, Y. Mao, et al., *Circulating Tumor DNA Mutation Profiling by Targeted Next Generation Sequencing Provides Guidance for Personalized Treatments in Multiple Cancer Types.* Sci Rep, 2017. **7**(1): p. 583.

8. H. Wang, Z.W. Li, Q. Ou, X. Wu, M. Nagasaka, Y. Shao, et al., *NTRK fusion positive colorectal cancer is a unique subset of CRC with high TMB and microsatellite instability.* Cancer Med, 2022. **11**(13): p. 2541-2549.

9. A.M. Bolger, M. Lohse and B. Usadel, *Trimmomatic: a flexible trimmer for Illumina sequence data.* Bioinformatics, 2014. **30**(15): p. 2114-20.

10. C. Zhu, L. Zhu, Y. Gu, P. Liu, X. Tong, G. Wu, et al., *Genomic Profiling Reveals the Molecular Landscape of Gastrointestinal Tract Cancers in Chinese Patients.* Front Genet, 2021. **12**: p. 608742.

11. T. Li, J. Fu, Z. Zeng, D. Cohen, J. Li, Q. Chen, et al., *TIMER2.0 for analysis of tumor-infiltrating immune cells.* Nucleic Acids Res, 2020. **48**(W1): p. W509-W514.

12. E. Becht, N.A. Giraldo, L. Lacroix, B. Buttard, N. Elarouci, F. Petitprez, et al., *Estimating the population abundance of tissue-infiltrating immune and stromal cell populations using gene expression.* Genome Biol, 2016. **17**(1): p. 218.

13. V. Thorsson, D.L. Gibbs, S.D. Brown, D. Wolf, D.S. Bortone, T.H. Ou Yang, et al., *The Immune Landscape of Cancer.* Immunity, 2018. **48**(4): p. 812-830 e14.

14. A. Liberzon, A. Subramanian, R. Pinchback, H. Thorvaldsdottir, P. Tamayo and J.P. Mesirov, *Molecular signatures database (MSigDB) 3.0.* Bioinformatics, 2011. **27**(12): p. 1739-40.

15. A. Subramanian, H. Kuehn, J. Gould, P. Tamayo and J.P. Mesirov, *GSEA-P: a desktop application for Gene Set Enrichment Analysis.* Bioinformatics, 2007. **23**(23): p. 3251-3.

## Supplementary Tables

| **Table S1. *DOT1L* mutations identified in the study cohort** | | | | | | | |
| --- | --- | --- | --- | --- | --- | --- | --- |
| Patient | Exon | AA Change | Start position | End position | Ref | Alt | Mutation type |
| 4 | Exon5 | c.286A>G (p.M96V) | chr19:2191032 | chr19:2191032 | A | G | Missense |
| 5 | Exon5 | c.409G>A (p.G137R) | chr19:2191155 | chr19:2191155 | G | A | Missense |
| 6 | Exon7 | c.623G>T (p.W208L) | chr19:2194548 | chr19:2194548 | G | T | Missense |
| 1 | Exon14 | c.1183C>T (p.R395C) | chr19:2210686 | chr19:2210686 | C | T | Missense |
| 8 | Exon14 | c.1184G>T (p.R395L) | chr19:2210687 | chr19:2210687 | G | T | Missense |
| 7 | Exon14 | c.1259C>T (p.A420V) | chr19:2210762 | chr19:2210762 | C | T | Missense |
| 3 | Exon21 | c.2471A>C (p.K824T) | chr19:2217016 | chr19:2217016 | A | C | Missense |
| 10 | Exon21 | c.2507G>T (p.G836V) | chr19:2217052 | chr19:2217052 | G | T | Missense |
| 3 | Exon24 | c.3319C>T (p.R1107C) | chr19:2222487 | chr19:2222487 | C | T | Missense |
| 9 | Exon25 | c.3446C>T (p.S1149F) | chr19:2223335 | chr19:2223335 | C | T | Missense |
| 11 | Exon14 | c.1199delA (p. N400Tfs*18) | chr19:2210700 | chr19:2210700 | A | - | Frameshift deletion |
| 12 | Exon15 | c.1421dupC (p. L475Afs*79) | chr19:2211164 | chr19:2211165 | - | C | Frameshift insertion |
| 12 | Exon15 | c.1422delGinsCT (p. L475Sfs*79) | chr19:2211168 | chr19:2211168 | G | CT | Frameshift insertion |
| 13 | Exon22 | c.2578delG (p. E860Sfs*208) | chr19:2217801 | chr19:2217801 | G | - | Frameshift deletion |
| 14 | Exon24 | c.3309delG (p. S1104Pfs*47) | chr19:2222477 | chr19:2222477 | G | - | Frameshift deletion |
| 2 | Exon22 | c.2683G>T (p.E895*) | chr19:2217909 | chr19:2217909 | G | T | Nonsense |
| AA, amino acid; chr, chromosome; Ref, reference allele; Alt, variant allele | | | | | | | |

| **Table S2. Clinical characteristics of patients in the NSCLC-ICI cohort (N=349)** | | | | |
| --- | --- | --- | --- | --- |
| Characteristic | All  (N=349) | *DOT1L*-WT  (N=336) | *DOT1L*-MUT  (N=13) | *P* value |
| Age |  |  |  | 0.77 |
| <60 | 116 (33.2%) | 111 (33.0%) | 5 (38.5%) |  |
| ≥60 | 233 (66.8%) | 225 (67.0%) | 8 (61.5%) |  |
| Sex |  |  |  | 0.57 |
| Female | 178 (51.0%) | 170 (50.6%) | 8 (61.5%) |  |
| Male | 171 (49.0%) | 166 (49.4%) | 5 (38.5%) |  |
| Smoking history |  |  |  | 0.48 |
| Never smokers | 68 (19.5%) | 67 (19.9%) | 1 (7.69%) |  |
| Current/former smokers | 281 (80.5%) | 269 (80.1%) | 12 (92.3%) |  |
| Histology |  |  |  | 0.70 |
| Squamous | 54 (15.5%) | 53 (15.8%) | 1 (7.69%) |  |
| Non-squamous | 295 (84.5%) | 283 (84.2%) | 12 (92.3%) |  |
| Treatment |  |  |  | >0.99 |
| Monotherapy | 240 (68.8%) | 231 (68.8%) | 9 (69.2%) |  |
| Combination therapy | 109 (31.2%) | 105 (31.2%) | 4 (30.8%) |  |
| Best RECIST Distribution |  |  |  | <0.001 |
| CR | 7 (2.01%) | 5 (1.49%) | 2 (15.4%) |  |
| PR | 78 (22.3%) | 71 (21.1%) | 7 (53.8%) |  |
| SD | 120 (34.4%) | 118 (35.1%) | 2 (15.4%) |  |
| PD | 137 (39.3%) | 135 (40.2%) | 2 (15.4%) |  |
| Unknown | 7 (2.01%) | 7 (2.08%) | 0 (0.00%) |  |
| Durable Clinical benefit |  |  |  | 0.02 |
| DCB | 120 (34.4%) | 111 (33.0%) | 9 (69.2%) |  |
| NCB | 208 (59.6%) | 204 (60.7%) | 4 (30.8%) |  |
| Unknown | 21 (6.02%) | 21 (6.25%) | 0 (0.00%) |  |
| TPS |  |  |  | 0.35 |
| <50% | 145 (41.5%) | 138 (41.1%) | 7 (53.8%) |  |
| ≥50% | 41 (11.7%) | 41 (12.2%) | 0 (0.00%) |  |
| Unknown | 163 (46.7%) | 157 (46.7%) | 6 (46.2%) |  |
| TMB status |  |  |  | 0.005 |
| TMB-L (＜10 muts/Mb) | 239 (68.5%) | 235 (69.9%) | 4 (30.8%) |  |
| TMB-H (≥10 muts/Mb) | 110 (31.5%) | 101 (30.1%) | 9 (69.2%) |  |
| CR, complete response; PR, partial response; SD, stable disease; PD, progressive disease; DCB, durable clinical benefit; NCB, no clinical benefit; TPS, tumor proportion score | | | | |

| **Table S3. Univariate and multivariate analyses of risk factors in the NSCLC-ICI cohort.** | | | | |
| --- | --- | --- | --- | --- |
| Risk factor | Univariate analysis | | Multivariate analysis | |
|  | HR (95% CI) | *P*-value | HR (95% CI) | *P*-value |
| Age (≥60 year vs. <60 years) | 1.04 (0.80-1.34) | 0.767 | ~ | ~ |
| Sex (Male vs. Female) | 1.13 (0.89-1.44) | 0.308 | ~ | ~ |
| Smoking (Current/former vs. Never) | 0.69 (0.52-0.93) | **0.013** | 0.56 (0.37-0.84) | **0.006** |
| Histology (LUAD vs. Others) | 0.84 (0.60-1.18) | 0.325 | ~ | ~ |
| Treatment (Comb vs. Mono) | 0.55 (0.42-0.73) | **<0.001** | 0.43 (0.23-0.59) | **<0.001** |
| PD-L1 status (High vs. Low) | 0.47 (0.29-0.74) | **0.001** | 0.37 (0.21-0.56) | **<0.001** |
| TMB status (High vs. Low) | 0.64 (0.49-0.84) | **0.001** | 0.55 (0.36-0.85) | **0.007** |
| *LRP1B* (MUT vs. WT) | 0.43 (0.27-0.69) | **<0.001** | 0.79 (0.45-1.37) | 0.396 |
| *DOT1L* (MUT vs. WT) | 0.34 (0.15-0.76) | **0.006** | 0.17 (0.04-0.70) | **0.014** |
| Bold represents significant *P* values. | | | | |

| **Table S4. Gene set enrichment analysis in *DOT1L*-mutated versus *DOT1L*-WT patients.** | | | |
| --- | --- | --- | --- |
| Gene set | NES | *P*-value | *P*-adjust |
| HALLMARK_FATTY_ACID_METABOLISM | 1.673 | 0.003 | 0.014 |
| HALLMARK_E2F_TARGETS | 2.322 | 0.003 | 0.014 |
| HALLMARK_MYC_TARGETS_V1 | 1.707 | 0.003 | 0.014 |
| HALLMARK_G2M_CHECKPOINT | 2.401 | 0.003 | 0.014 |
| HALLMARK_MITOTIC_SPINDLE | 1.919 | 0.003 | 0.014 |
| HALLMARK_CHOLESTEROL_HOMEOSTASIS | 1.673 | 0.005 | 0.020 |
| HALLMARK_PROTEIN_SECRETION | 1.550 | 0.008 | 0.026 |
| HALLMARK_PI3K_AKT_MTOR_SIGNALING | 1.475 | 0.008 | 0.026 |
| HALLMARK_OXIDATIVE_PHOSPHORYLATION | 1.377 | 0.010 | 0.026 |
| HALLMARK_MTORC1_SIGNALING | 1.469 | 0.010 | 0.026 |
| HALLMARK_XENOBIOTIC_METABOLISM | 1.461 | 0.010 | 0.026 |
| HALLMARK_REACTIVE_OXYGEN_SPECIES_PATHWAY | 1.683 | 0.010 | 0.026 |
| HALLMARK_ADIPOGENESIS | 1.411 | 0.013 | 0.029 |
| HALLMARK_ESTROGEN_RESPONSE_EARLY | 1.301 | 0.026 | 0.052 |
| HALLMARK_UNFOLDED_PROTEIN_RESPONSE | 1.401 | 0.031 | 0.059 |
| HALLMARK_SPERMATOGENESIS | 1.296 | 0.041 | 0.073 |
| HALLMARK_INFLAMMATORY_RESPONSE | -2.017 | 0.001 | 0.012 |
| HALLMARK_TNFA_SIGNALING_VIA_NFKB | -2.155 | 0.001 | 0.012 |
| HALLMARK_INTERFERON_GAMMA_RESPONSE | -1.854 | 0.001 | 0.012 |
| HALLMARK_ALLOGRAFT_REJECTION | -2.026 | 0.001 | 0.012 |
| HALLMARK_EPITHELIAL_MESENCHYMAL_TRANSITION | -2.355 | 0.001 | 0.012 |
| HALLMARK_KRAS_SIGNALING_UP | -2.004 | 0.001 | 0.012 |
| HALLMARK_COMPLEMENT | -1.806 | 0.003 | 0.014 |
| HALLMARK_COAGULATION | -1.621 | 0.011 | 0.026 |
| HALLMARK_IL6_JAK_STAT3_SIGNALING | -1.893 | 0.011 | 0.026 |
| HALLMARK_IL2_STAT5_SIGNALING | -1.469 | 0.014 | 0.031 |
| HALLMARK_INTERFERON_ALPHA_RESPONSE | -1.604 | 0.017 | 0.036 |
| HALLMARK_ANGIOGENESIS | -1.482 | 0.041 | 0.073 |
| NES, normalized enrichment score | | | |

## Supplementary Figures


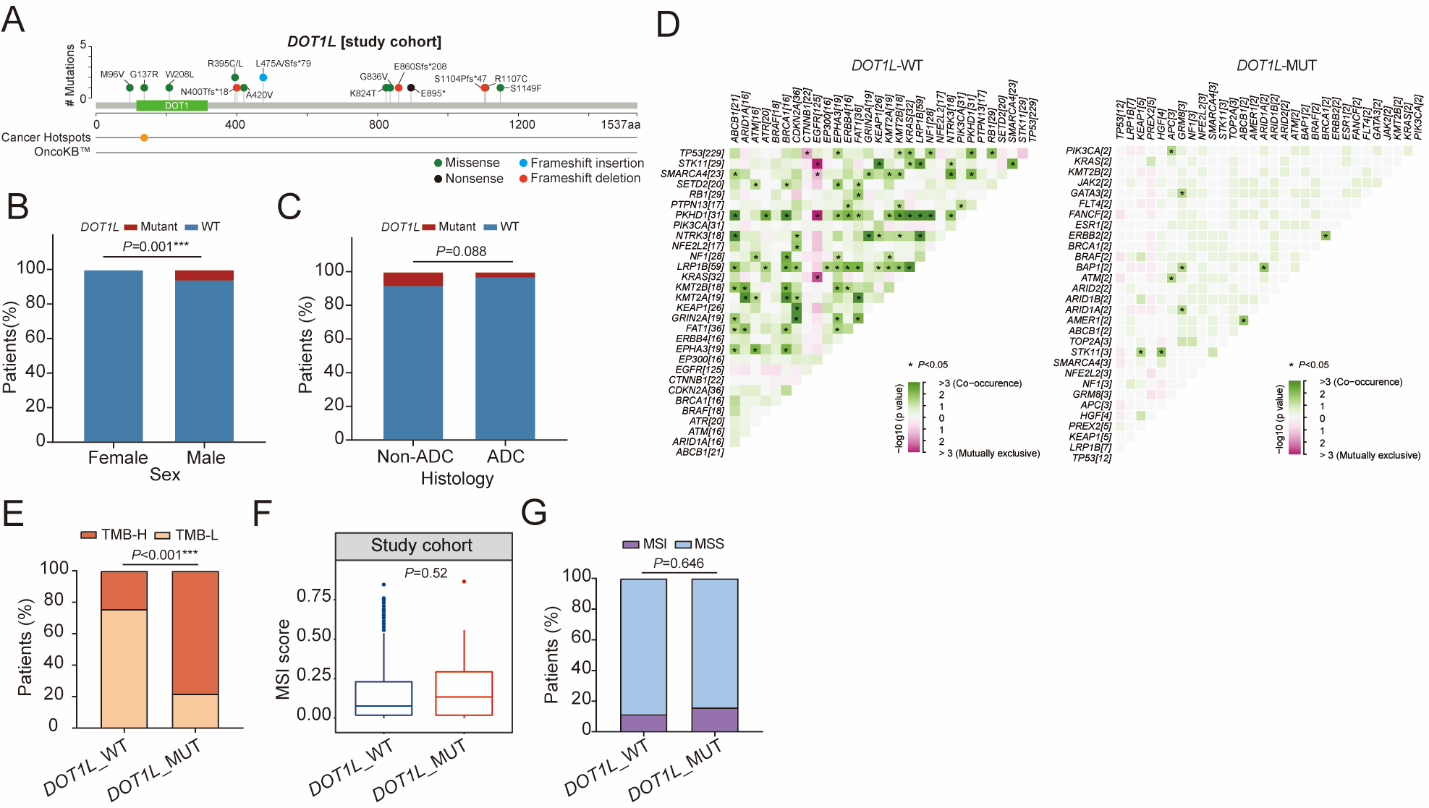


**Figure S1. Comparing the clinical and molecular features between *DOT1L­*-mutated and *DOT1L*-wildtype patients in the study cohort.**

**(A)** Lollipop plot showing the mutation sites of *DOT1L* in baseline tumor tissues of the study cohort. **(B, C)** Bar plot illustrating the distribution of *DOT1L*-mutated and *DOT1L*-wildtype patients between subgroup patients classified based on sex (B) and histology (C). **(D)** Heatmap showing the top 30 co-occurring and mutually exclusive genes in *DOT1L*-wildtype and *DOT1L*-mutated NSCLC patients in the study cohort. **(E)** Bar plot illustrating the distribution of TMB-high (≥10 Mut/Mb) and TMB-low (<10 Mut/Mb) patients in subgroup patients. **(F)** Box plot illustrating the microsatellite instability (MSI) score of subgroup patients. **(G)** Bar plot illustrating the distribution of MSI and microsatellite stable (MSS) patients in subgroup patients.


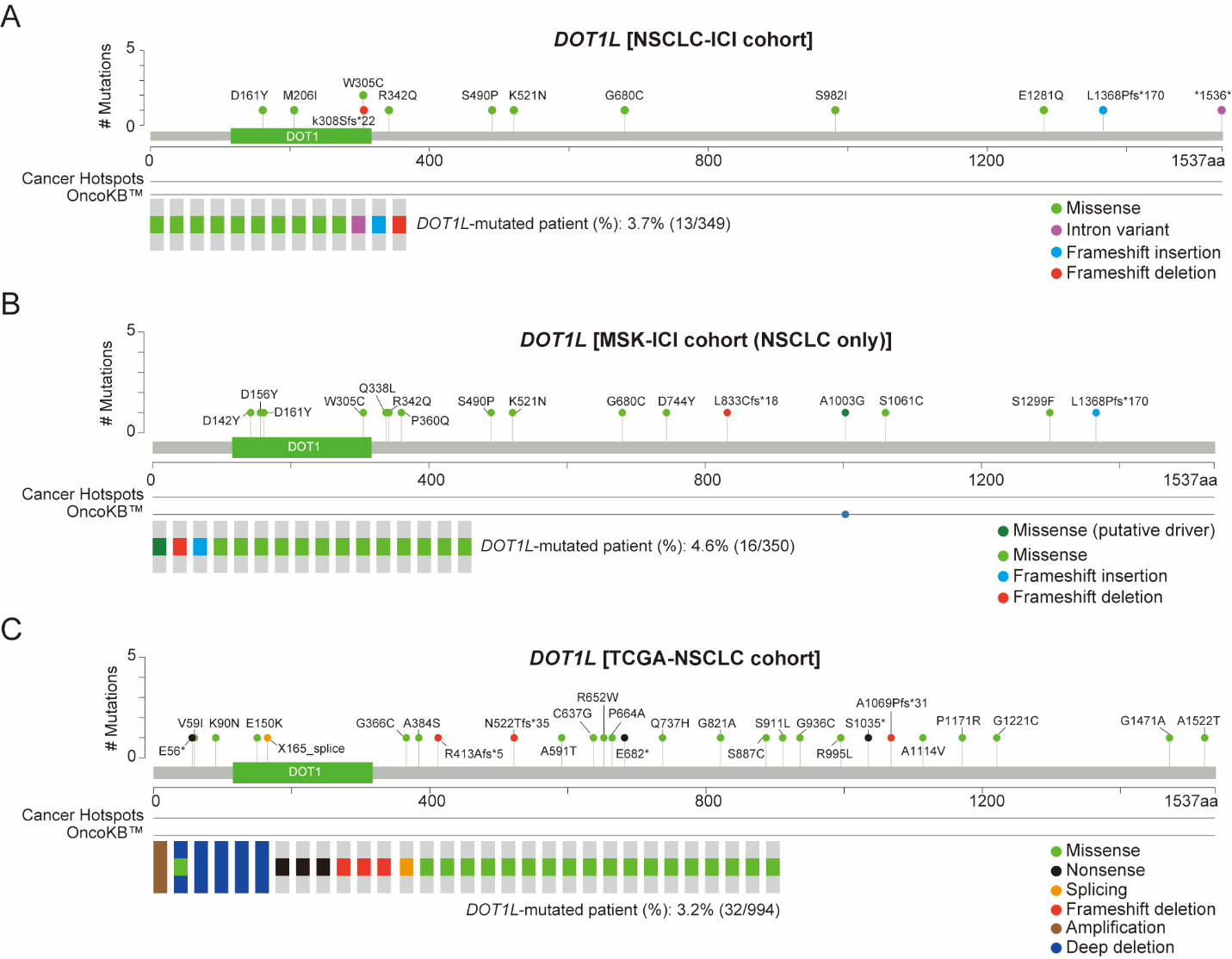


**Figure S2. *DOT1L* aberrations in external datasets.**

Lollipop plots illustrate the type and genomic location of *DOT1L* mutations identified within the NSCLC-ICI cohort (A), NSCLC patients in the MSK-ICI cohort (B), and the TCGA-NSCLC cohort (C).


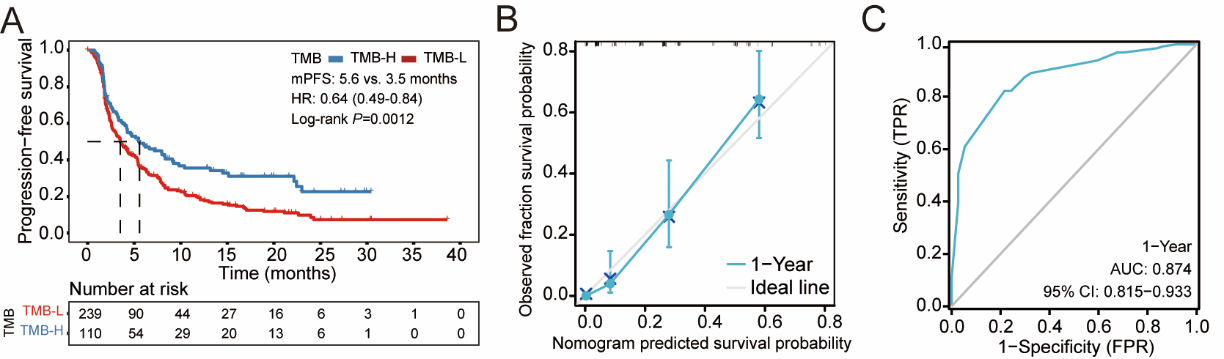


**Figure S3. Construction of a one-year survival probability model for progression risk using combined features from the NSCLC-ICI cohort.**

**(A)** Kaplan-Meier curves illustrate the progression-free survival of patients in strata of TMB status in the NSCLC-ICI cohort (N=349). **(B)** Comparison of the predicted one-year survival probability and observed fraction survival probability of NSCLC patients receiving ICI treatments. **(C)** Area under the curve plot of the predictive model based on significant risk factors identified in the multivariate analysis.
